# Draft Genome Sequence of the Extensively Drug-Resistant Pseudomonas aeruginosa Clinical Isolate TUEPA7472

**DOI:** 10.1128/MRA.01055-18

**Published:** 2018-09-27

**Authors:** Henrike Miess, Ghazaleh Jahanshah, Heike Brötz-Oesterhelt, Matthias Willmann, Silke Peter, Harald Gross

**Affiliations:** a Department of Pharmaceutical Biology, Institute of Pharmaceutical Sciences, University of Tübingen, Tübingen, Germany; b German Centre for Infection Research (DZIF), Partner Site Tübingen, Tübingen, Germany; c Microbial Bioactive Compounds, Interfaculty Institute of Microbiology and Infection Medicine Tübingen (IMIT), University of Tübingen, Tübingen, Germany; d Institute of Medical Microbiology and Hygiene, University of Tübingen, Tübingen, Germany; University of Maryland School of Medicine

## Abstract

Pseudomonas aeruginosa TUEPA7472 is extensively drug resistant (XDR) and is a representative Gram-negative rod that is multiresistant toward 4 classes of clinically relevant antibiotics (4MRGN). The 6.8-Mb draft genome sequence of this strain provides insight into these resistance mechanisms and the potential of the strain to produce virulence factors.

## ANNOUNCEMENT

Pseudomonas aeruginosa is an opportunistic pathogen that causes morbidity and mortality in humans with compromised natural defenses, and it is most prevalent among hospitalized patients with burn wounds, cystic fibrosis (1, 2), cancer, AIDS, or catheter treatment. The production of virulence factors (3, 4), biofilm formation (5), and its metabolic versatility allow for establishment and promotion of infection, while its intrinsic and recent emerging resistance to multiple antibiotics (6) makes treatment challenging.

Strain TUEPA7472 was recovered from a blood culture specimen at the Institute of Medical Microbiology and Hygiene in Tübingen, Germany. The isolate was resistant toward beta-lactams (piperacillin, piperacillin-tazobactam, cefepime, aztreonam, and meropenem), aminoglycosides (amikacin, gentamicin, and tobramycin), and fluoroquinolones (ciprofloxacin and levofloxacin) but remained susceptible toward colistin. Notably, the strain remained susceptible toward ceftazidime. Based on this antimicrobial susceptibility pattern, the strain can be classified as an extensively drug-resistant (XDR) bacterium (7) or, according to the current German national guideline (8, 9), as 4MRGN (multiresistant Gram-negative rod resistant toward 4 classes of clinically relevant antibiotics). Genomic DNA (gDNA) was extracted from 15 ml of an overnight culture grown in tryptic soy broth (TSB) at 30°C using the genomic DNA purification kit in combination with 100/G Genomic-tips (Qiagen), following the manufacturer´s protocol, except that the volumes handled in the first two steps were doubled. Aliquots were used to construct PacBio (2-kb insert size) and Illumina (TruSeq PCR-free kit) libraries. Sequencing on a PacBio RS II instrument yielded 113,538 subreads (142× coverage), with an *N*_50_ value of 12,864 bp, from two single-molecule real-time (SMRT) cells, while the 2 × 150-bp Illumina HiSeqX run produced 118,026,712 total reads (2,552× coverage), with a median length of 415 bp. The Illumina reads were filtered using BBDuk (BBMap suite version 36.77), and high-quality reads were subsequently assembled into contigs employing ABySS version 2.0.2 (10). The contigs were linked and placed into superscaffolds based on the alignment of the PacBio continuous long reads using BLASR (11) and SSPACE version 1.0 (12). Gapped regions within the superscaffolds were closed or reduced using the Illumina reads and GapFiller 1.10 (13). Finally, assembly errors and the nucleotide disagreements between the Illumina reads and scaffold sequences were corrected using Pilon version 1.21 (14). Software parameter settings were kept at the defaults. Overall, the hybrid *de novo* assembly resulted in a 6,806,824-bp nucleotide draft with a G+C content of 66.2%, which consisted of 18 scaffolds representing one chromosome. One scaffold had a single additional gap that was estimated to be 629 bp. The assembled contigs were annotated with the Prokaryotic Genome Annotation Pipeline (PGAP) (15), resulting in the annotation of 6,403 coding sequences.

Automated bioinformatics analyses (16, 17) predicted 12 biosynthetic gene clusters coding for secondary metabolites. Six of these matched known clusters for pyoverdin, a pyrrolizidine, pyochelin, pyocyanin, 2× homoserine-lactone, and 2-amino-4-methoxy-*trans*-3-butenoic acid. The remaining clusters were predicted to encode 1 nonribosomal peptide synthetase (NRPS), 1 hybrid NRPS-polyketide synthase (NRPS-PKS), and 3 ribosomally synthesized and posttranslationally modified peptide (RiPP)-based compounds.

By using ResFinder 3.0 (18), several antibiotic resistance genes were found in this genome, including the beta-lactamase resistance genes *bla*_PAO_ and *bla*_OXA-50_, the aminoglycoside resistance gene *aph(3′)-IIb*, the fluoroquinolone resistance gene *crpP*, and the fosfomycin resistance gene *fosA*.

### Data availability.

This whole-genome sequencing (WGS) project has been deposited at DDBJ/ENA/GenBank under the accession number QOLE00000000. The version described in this paper is version QOLE01000000. The sequencing reads have been deposited under the accession number SRP156998. All reads have been deposited to the SRA and are associated with BioProject number PRJNA475453.
